# Deep Learning-Based Pointer Meter Reading Recognition for Advancing Manufacturing Digital Transformation Research

**DOI:** 10.3390/s25010244

**Published:** 2025-01-03

**Authors:** Xiang Li, Jun Zhao, Changchang Zeng, Yong Yao, Sen Zhang, Suixian Yang

**Affiliations:** 1 School of Mechanical Engineering, Sichuan University, Chengdu 610065, China; xiangli@stu.scu.edu.cn; 2 School of Computer Science, Civil Aviation Flight University of China, Guanghan 618307, China; 3 National Institute of Measurement and Testing Technology, Chengdu 610056, China; zhaojun@nimtt.com (J.Z.); yaoyong@nimtt.com (Y.Y.); 4 School of Big Data, Guizhou Institute of Technology, Guiyang 550003, China; sen.zhang@git.edu.cn

**Keywords:** PMRR, deep learning, digital transformation, image processing, pattern recognition

## Abstract

With the digital transformation of the manufacturing industry, data monitoring and collecting in the manufacturing process become essential. Pointer meter reading recognition (PMRR) is a key element in data monitoring throughout the manufacturing process. However, existing PMRR methods have low accuracy and insufficient robustness due to issues such as blur, uneven illumination, tilt, and complex backgrounds in meter images. To address these challenges, we propose an end-to-end PMRR method based on a decoupled circle head detection algorithm (YOLOX-DC) and a Unet-like pure Transformer segmentation network (PM-SwinUnet). First, according to the characteristics of the pointer dial, the YOLOX-DC detection algorithm is designed based on the exceeding you only look once detector (YOLOX). The decoupled circle head of YOLOX-DC detects the pointer meter dial more accurately than the commonly used rectangular detection head. Second, the window multi-head attention of the PM-SwinUnet network enhances the feature extraction ability of pointer meter images and solves problems of missed scale detection and incomplete pointer segmentation. Additionally, the scale and pointer fitting module is introduced into the PM-SwinUnet to locate the accurate position of the scale and pointer. Finally, through the angle relationship between the pointer and the first two main scale lines, the pointer meter reading is accurately calculated by the improved angle method. Experimental results demonstrate the effectiveness and superiority of the proposed end-to-end method across three-pointer meter datasets. Furthermore, it provides a rapid and robust approach to the digital transformation of manufacturing systems.

## 1. Introduction

The core of digital transformation in the manufacturing industry lies in the acquisition of real-time data during the manufacturing process. Pointer meters are widely used in industrial processes to monitor equipment status due to their strong resistance to electromagnetic interference and low cost. However, a prominent challenge is accurately identifying the readings of pointer meters [1]. Manual reading is often unsuitable in extreme environments such as radiation, high temperature, or high pressure. With advancements in machine vision, inspection robots, and industrial cameras, various automatic identification methods for pointer meter readings have been proposed [2,3,4,5]. Most of these methods rely on traditional step-by-step image processing techniques, which are less robust when dealing with images affected by blur, exposure, darkness, or tilt. Therefore, the intelligent recognition of pointer meter reading in complex industrial scenes has important practical and research value and has garnered increasing attention from researchers.

Intelligent recognition pointer meter reading involves two steps: dial detection and reading recognition. Dial detection employs machine vision or image processing techniques to locate the dial within the meter image. Common dial detection methods include the Hough transform [6,7] and template matching [8,9]. However, these approaches often exhibit poor accuracy in images with complex backgrounds, and template matching requires pre-prepared features, limiting its flexibility and robustness. Recently, deep learning technology has been successfully applied to medical imaging [10], remote sensing [11], agriculture [12] and marine life detection [13]. Consequently, some researchers have applied deep learning to pointer meter dial detection [14,15,16,17]. Fan et al. [14] proposed an improved YOLOv5-based model to enhance meter dial detection accuracy. Zhou et al. [15] used a two-stage convolutional neural network (CNN) to extract the meter reading area. Salomon et al. [17] used YOLOv4 to directly detect dashboards within input images. Compared to these deep learning methods, YOLOX [18] adopts an anchor-free method for faster detection, which improves computational efficiency compared to anchor-based methods. Additionally, it incorporates the advanced label assignment strategy SimOTA to enhance detection accuracy. Therefore, this paper proposes the YOLOX-DC dial detection algorithm based on YOLOX. This algorithm employs a decoupled circular detection head to identify pointer meter dials. Compared with rectangular detection heads, decoupled circular detection heads detect meter dials more accurately and contain less background information.

Pointer meter reading recognition (PMRR) involves first determining the positions of the scale lines and pointer, thereby establishing a mapping relationship between the pointer’s deflection angle and the meter reading [3,19]. The pointer meter reading is then calculated using either the angle method or the distance method. For pointer meter images with complex backgrounds, traditional image preprocessing techniques [20,21,22] are complicated and lack robustness. Recently, researchers have applied image segmentation technology to PMRR [23,24]. Wan et al. [24] utilized U-Net to extract the scale marks and pointers of pointer meters, achieving automatic reading based on U-Net segmentation results. However, the U-Net network tends to lose boundary information of small-scale targets during image segmentation. Since the introduction of the Swin Transformer [25] into computer vision, advanced image segmentation models like Swin-Unet [26,27] and Ds-TransUNet [28] have been developed. The window multi-head attention in Swin Transformer enhances focus on small and long objectives. When combined with the U-Net architecture, this significantly improves the network’s ability to extract features from images. Therefore, this paper proposes a PM-SwinUnet image segmentation model to extract meter elements from the dial image. The model incorporates a Scale and Pointer Fitting Module to accurately locate the scale and pointer. Finally, an improved angle method is used to accurately identify pointer meter readings. The main contributions of this paper can be summarized as follows.

1. To enhance the accuracy of pointer meter dial detection, the YOLOX-DC algorithm with a decoupled circle head for the pointer dial’s characteristics is proposed. Compared to the rectangular detection head algorithm, the YOLOX-DC algorithm with a decoupled circle head significantly improves the detection accuracy of pointer meter dials. The detected dial images provide data support for the PMRR task.
2. To enhance the feature extraction capability of the image segmentation network, this paper proposes the PM-SwinUnet network, which is based on the Swin Transformer model and follows a UNet-like architecture. The PM-SwinUnet network effectively addresses the issues of missed scale detection and incomplete pointer segmentation. By integrating the Scale and Pointer Fitting Module, the network achieves precise localization of scales and pointers.
3. The angle relationship of the pointer relative to the first two main scale lines is utilized to achieve accurate readings through the improved angle method. The superiority and robustness of the proposed end-to-end method are verified by extensive experiments under various types of pointer meters and different scenarios.

## 2. Related Work

### 2.1. Dial Detection

Current dial detection methods are divided into two major categories: rule-based methods and statistical-based methods. Rule-based methods [8,9,21,22,29] rely on prior knowledge and manually extracted features, such as meter range, dial circumference, and pointer characteristics. Mo et al. [9] performed a pointer meter reading based on template matching. However, this method requires pre-collection of template images for various meters, limiting its flexibility and universality. Devyatkin et al. [29] extracted the region of interest using the Otsu algorithm and then detected pointers using the Hough algorithm. While effective for pointer extraction, this method is prone to significant errors when shadows fall on the pointer at certain angles. Rule-based methods are limited in complex environments because they rely on prior knowledge or inherent meter characteristics and require extensive calculations. Therefore, many researchers have proposed statistical-based methods. These methods can automatically learn image features from numerous images, with deep learning methods being widely applied for pointer meter readings [23,24,30,31,32,33]. Hou et al. [30] proposed a pointer meter recognition method using wireless sensor networks and a lightweight CNN. Ji et al. [33] developed a calibration method for circular pointer meters based on the YOLOv5s network, which detects scale values in the meter panel as key points. Compared to manually designed feature extraction methods, deep learning methods can extract deep image information from images. However, these deep learning-based methods use rectangular detection heads to detect pointer meter dials, resulting in poor generalization and robustness under conditions of uneven illumination, significant lighting variations, and image blur.

### 2.2. Dial Image Segmentation

The accuracy of automatic pointer meter reading recognition largely depends on the precise detection of the pointer and scale marks. Therefore, it is necessary to accurately extract meter scales, pointers, and scale values from the dial image. Traditional methods used threshold segmentation [34], edge detection [35], and straight-line detection [36] to fit dial and pointer line equations. These methods require manual parameter settings for each image. When meter images are affected by lighting variations or external occlusions, the preset parameters often fail, reducing their effectiveness. With the development of segmentation technology, Zuo et al. [23] utilized Mask R-CNN to segment meter and pointer areas, followed by the PCA algorithm to fit the pointer line. However, the recognition speed is slower, and compared with Mask R-CNN, U-Net only requires fewer training samples to complete accurate segmentation. Wan et al. [24] employed U-Net to extract scale marks and pointers from pointer meters, enabling automatic reading through U-Net segmentation results and scale line contour fitting. However, the U-Net network easily loses boundary information for small-scale targets during image segmentation. Moreover, due to the convolution kernel characteristics of CNN, these methods cannot extract global features and long-dimensional features from the pointer meter image, resulting in incomplete segmentation of longer pointers. Zhang et al. [27] addressed this limitation by applying the Swin Transformer segmentation network to generate mask images of scales and pointers, followed by the minimum skeleton circle method to fit the pointer line equation. While this approach improves feature extraction, it is time-consuming and unsuitable for practical applications.

### 2.3. Reading Recognition

Currently, methods for recognition of pointer meter readings include the distance method [14], the angle method [2,27,32,37,38], and the template method [39]. Fan et al. [14] proposed converting the arc-shaped dial into a rectangular dial and transforming the arc-shaped scale into a straight line, calculating the reading by measuring the pointer and scale distance. Hou et al. [32] adopted an enhanced angle method that uses the rotation angle of the pointer to the two nearest tick marks to calculate the reading. Zhang et al. [38] proposed a deep regression network to predict the meter pointer rotation angle and calculate the reading based on the angle method. Hassan et al. [39] designed a template-matching approach using an imaginary template consisting of concentric circles with radius lines at specific intervals to determine the pointer’s value. From the above research, it can be concluded that the distance method causes information loss when converting the arc-shaped dial into a straight line, while the template method requires pre-prepared template meters. Therefore, the angle method is more competitive than the distance and template methods. However, the angle method requires multiple angles and scale values to calculate meter readings. First, it increases the computational load of the segmentation network and recognition algorithm. Second, the more parameters the recognition algorithm has, the greater the cumulative error.

To address these problems, this article proposes an end-to-end pointer meter reading recognition method based on YOLOX-DC and PM-SwinUnet. This method requires only two angles and a scale value to calculate the reading. In special circumstances, it can accurately compute the pointer meter reading using just two angles, significantly reducing both the computational load and the error of the recognition algorithm.

## 3. Materials and Methods

**Figure 1:**
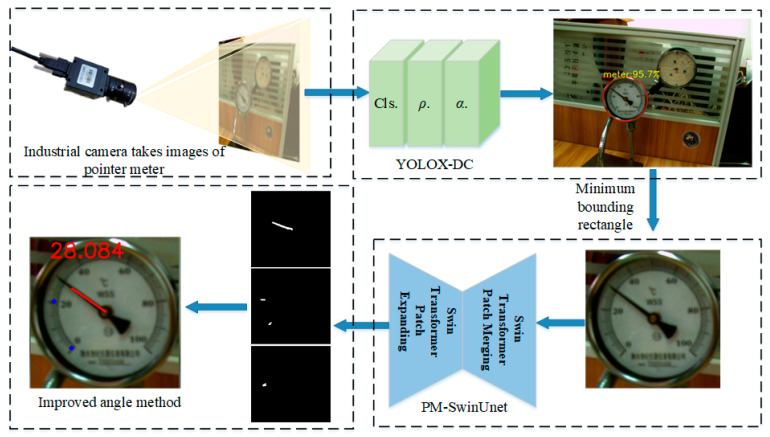
The framework of the proposed pointer meter reading recognition method is based on deep learning. “meter: 95.7%”: meter represents the category and 95.7% represents the confidence.

The framework of our method is shown in Figure 1. The pointer meter images are collected by an industrial camera. First, the YOLOX-DC dial detection algorithm detects the dial within the pointer meter image. Next, the PM-SwinUnet image segmentation network simultaneously segments the pointer, key scales, and scale readings from the dial image. Finally, based on the segmentation results, the skeleton refinement and center point extraction algorithms are applied to remove edge pixels, which form the pointer skeleton and scale’s center point coordinates at the unit pixel. Furthermore, the improved angle method is employed to compute the final pointer meter reading. The specific components of the algorithm are detailed in the following sections.

### 3.1. YOLOX-DC Dial Detection

**Figure 2:**
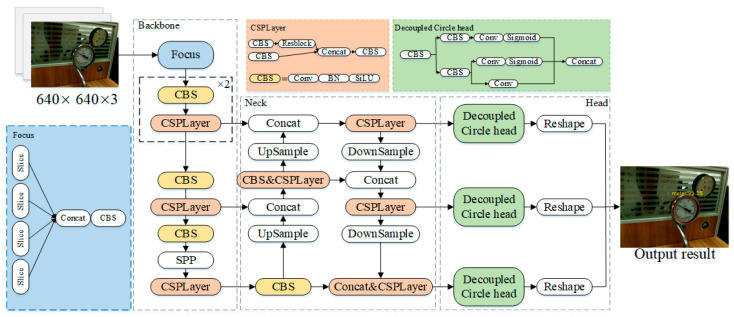
Architecture of YOLOX-DC model.

Inspired by the YOLOX algorithm [18], this paper proposes the YOLOX-DC algorithm. The structure of the YOLOX-DC algorithm is illustrated in Figure 2. The YOLOX-DC network is primarily composed of three components: the backbone, neck, and detection head. The backbone network uses the CSPDarkNet structure, where the Focus module downsamples the feature map, and the SPP module expands the receptive field. Between these modules, multiple stacked CBS modules and CSP layers are employed to transmit and extract features effectively. In the detection head stage, YOLOX-DC uses the Decoupled Circle head for classification and regression tasks. Designed to align with the characteristics of the pointer meter dial, the Decoupled Circle head enhances the model’s learning ability for dial-specific targets, resulting in a more robust and accurate pointer meter detection model.

**Figure 3:**
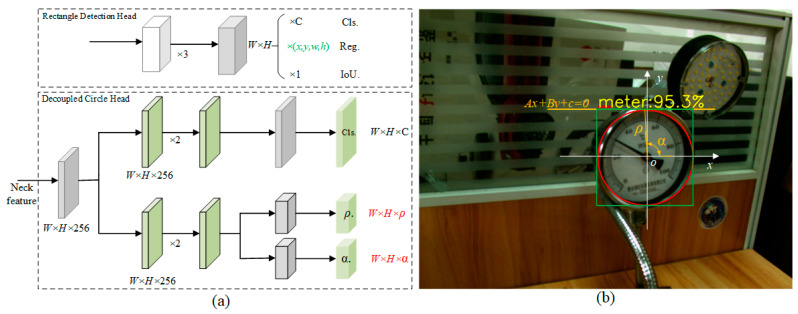
(**a**) The difference between the decoupled circle detection head and rectangular detection head, (**b**) Schematic diagrams of the two types of detection heads, red is the decoupled circle detection head, and green is the rectangular detection head.

The most intuitive difference between the proposed Decoupled Circle Head and the rectangular detection head lies in the representation of the bounding box, as shown in the Figure 3a. The common detection method is the rectangular bounding box, which requires learning the four parameters *x*, *y*, *w*, and *h* to locate the detection target. In contrast, the circular edge of the pointer meter dial boundary can be described using a parametric circular equation. The proposed Decoupled Circle Head adopts polar coordinates to decouple the pointer dial contour and requires learning only two parameters, *ρ* and *α,* to locate the dial position. For example, Figure 3b illustrates the circular edge equation relative to the current position. The current position is treated as the origin of the Cartesian coordinate system. The required training parameters are reduced. In general, the circular equation can be expressed as follows.

(1) $${\left(x-a\right)}^{2}+{\left(y-b\right)}^{2}=r^{2},$$

where (*a*, *b*) is the center of the circle at the current dial position and r is the radius. The physical meaning of these parameters in the image is vague, which is not conducive to network learning. Therefore, we convert the above circle equations into polar coordinate equations. According to the relationship between the polar coordinates of the circle and the Cartesian coordinates, the x=ρcosα, y=ρsinα. Substituting these expressions into Equation (1), the polar equation of the circle can be obtained as follows.

(2) $${\left(\rho \cos\alpha -a\right)}^{2}+{\left(\rho \cos\alpha -b\right)}^{2}=r^{2},$$

To simplify the calculation, we introduce a line Ax+By+C=0 intersecting the circle, where *A*, *B*, and *C* are the three parameters. The equation of the line is transformed into the polar coordinate equation $\rho_{L}=x\cos\alpha +y\sin\alpha$. As shown in Figure 3b, $\rho_{L}$ is the nearest distance between the origin (current location) and line equation, *α* is the rotation angle from the *x*-axis positive direction to above nearest direction. Assuming that the center of the circle is the origin of the coordinate system, it is worth noting that $\rho_{L}=\rho$. Therefore, when solving, we can obtain the polar diameter *ρ* and polar angle *α* of the circle equation from the line equation. In addition, we provide the calculation process for *ρ* and *α*, as shown in Algorithm 1.
**Algorithm 1:** Circular Boundary Label Generation1.    For (*x_i_, y_i_*), *i* = 1, 2, 3, 2.            Calculate the current position point (*x*_0_, *y*_0_). 3.            Obtain the circular boundary information based on Equation (1). 4.            Derive Equation (2) based on the relationship between polar coordinates and Cartesian coordinates. 5.            Introduce a line intersecting with the circle. 6.            Obtain *ρ* based on the point-to-line distance formula. 7.            Calculate the angle between the line and the positive direction of the *x*-axis to obtain *α*. 8.            If the perpendicular direction points below the *x*-axis, adjust the angle *α*. 9.    End 10.  Compare the values of *ρ*, and the pair (*ρ*, *α*) with the maximum *ρ* is chosen as the final label for the circle.

According to the above analysis, the loss function for the model can be expressed as follows.

(3) $$L=\lambda_{cls}L_{cls}+\lambda_{\rho }L_{\rho }+\lambda_{\alpha }L_{\alpha },$$

(4) $$L_{cls}=-\frac{1}{\left|N\right|}\sum_{i\in N}\left[y_{i}\log{\hat{y}}_{i}+(1-y_{i})\log(1-{\hat{y}}_{i})\right],$$

(5) $$L_{\rho }=-\frac{1}{N}\sum_{i}\log\frac{\max({\hat{\rho }}_{i},\rho_{i})-\min({\hat{\rho }}_{i},\rho_{i})}{\max({\hat{\rho }}_{i},\rho_{i})},$$

(6) $$L_{\alpha }=1-\frac{1}{N}\sum_{i}\cos\frac{\left|{\hat{\alpha }}_{i}-\alpha_{i}\right|}{2},$$

where *L_cls_* represents the reading/non-reading region classification loss, *L_ρ_* and *L_α_* represent the regression loss of line parameters *ρ* and *α* in polar coordinates, respectively, and λ*_cls_*, λ*_ρ_* and λ*_α_* are used to weigh the importance between the three losses. In this experiment, we set the three parameters uniformly to 1.

### 3.2. PM-SwinUnet Image Segmentation Network

Currently, methods for obtaining meter pointers, scales, and scale values include threshold segmentation, edge detection, and image segmentation. Threshold segmentation and edge detection methods require pre-designed parameters, making them unsuitable for meter images with complex backgrounds in industrial environments. Similarly, CNN-based image segmentation methods are limited by the convolutional kernel’s characteristics, which hinder the extraction of global features and long-dimensional information from pointer meter images. This often leads to missed scale detections and incomplete pointer segmentations. To address these shortcomings, this paper proposes a pointer meter image segmentation model based on a UNet-like Swin Transformer, which simultaneously segments the three elements of the pointer meter image.

**Figure 4:**
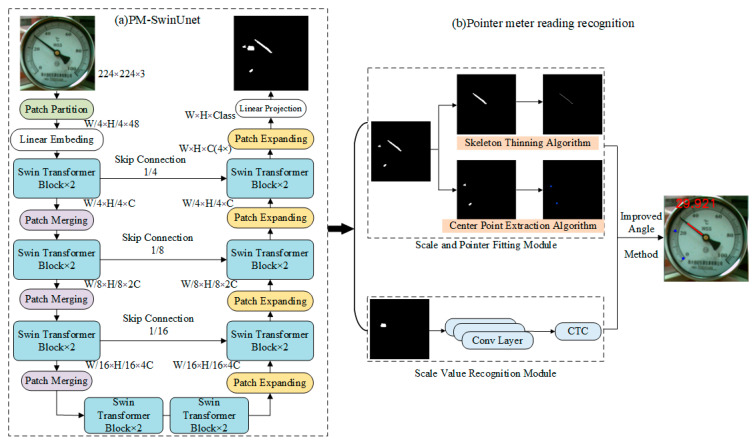
The pointer meter reading recognition method is based on the PM-SwinUnet network.

**Figure 5:**
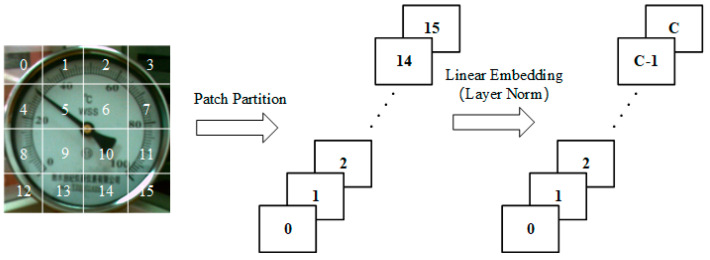
Schematic representation of patch partition and linear embedding for meter images.

The overall architecture of PM-SwinUnet is illustrated in Figure 4a. PM-SwinUnet consists of encoders, decoders, bottleneck connections, and skip connections, with its basic unit being the Swin Transformer block [25]. To convert the image into a sequence input, the encoder divides the pointer meter image into non-overlapping patches of size 4 × 4. Through this division method, the feature dimension of each patch becomes 4 × 4 × 3 = 48. Additionally, each patch is converted by a linear embedding layer to obtain a patch token; the above process is illustrated in Figure 5. Then, through the combination of Swin Transformer block and patch merging, the feature dimension 48 is mapped to an arbitrary dimension C. When Swin Transformer calculates the meter image features, it adds the previous layer’s window features to the next layer’s window to compute the global features of the image. This approach effectively addresses the issue of scattered pointer pixel areas distributed over a long region of the meter image. Inspired by the U-Net network structure [40], a symmetric decoder based on the Swin Transformer is designed. The decoder consists of Swin Transformer blocks and patch-expanding layers. The expansion layers upsample adjacent feature maps to double the resolution while fusing the contextual features extracted by the decoder’s Swin Transformer blocks with the multi-scale features of the encoder via skip connections. This fusion compensates for information loss caused by downsampling. Finally, the expansion layers perform four rounds of upsampling until the feature resolution matches the input image resolution. After a linear projection of the upsampled features, the segmentation results of the meter image elements are generated.

**Figure 6:**
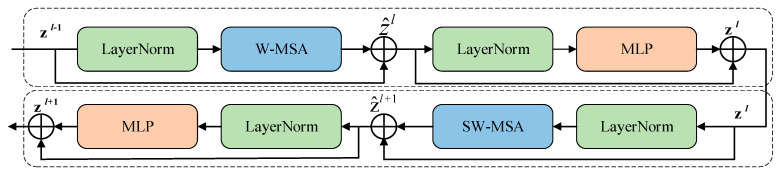
Structure of Swin Transformer block.

The Swin Transformer block is based on the concept of a moving window, which differs from the traditional multi-head self-attention (MSA) module. Each Swin Transformer block consists of multi-head self-attention, a LayerNorm (LN) layer, residual connections, and an MLP. Figure 6 shows a schematic diagram of two consecutive Swin Transformer blocks. In the two consecutive Transformer blocks, the window-based multi-head self-attention (W-MSA) module and the shift window-based multi-head self-attention (SW-MSA) module are utilized. This window division mechanism enables the Swin Transformer block to effectively capture both local and global features. Moreover, by integrating the Swin Transformer features into the decoder stage of U-Net, PM-SwinUnet effectively combines multi-scale contextual information with detailed local features. This fusion helps the model focus on small objects while preserving their boundaries and details. The mathematical representation of the Swin Transformer block can be expressed as follows:

(7) $${\hat{z}}^{l}=W\text{-}MSA(LN(z^{l-1}))+z^{l-1},$$

(8) $$z^{l}=MLP(LN({\hat{z}}^{l}))+{\hat{z}}^{l},$$

(9) $${\hat{z}}^{l+1}=SW\text{-}MSA(LN(z^{l}))+z^{l},$$

(10) $$z^{l+1}=MLP(LN({\hat{z}}^{l+1})+{\hat{z}}^{l+1},$$

where ${\hat{z}}^{l}$ and z*^l^* represent the outputs of the *l*th block (S)W-MSA module and MLP module, respectively. Similar to previous work [41], self-attention is calculated as follows.

(11) $$Attention(Q,K,V)=Softmax(\frac{QK^{T}}{\sqrt{d}}+B)V,$$

where *Q*, *K*, and *V* represent the query matrix, key matrix and value matrix, respectively. *B* represents the bias matrix.

### 3.3. Scale and Pointer Fitting Module

In pointer meter images, the scale positions are located on the circle of rotation of the pointer. Therefore, the relationship between the pointer and the scale serves as the foundation for identifying the meter reading. The three-meter elements of pointer, scale and scale reading have been segmented by PM-SwinUnet. But their exact positions are yet to be determined. Mask maps are first generated through the PM-SwinUnet segmentation network to accurately locate the positions of the pointer and scale. The Scale and Pointer Fitting Module then processes these mask maps to determine the pointer and scale positions using the skeleton thinning algorithm and the center point extraction algorithm, respectively, as shown in Figure 4b. Positioning the meter pointer involves determining both its position and direction. The specific steps for determining the pointer position using the Skeleton Thinning algorithm are as follows.

(1) PM-SwinUnet was used to segment the pointer image, resulting in a mask image containing only the pointer area. Since the pointer pixel region is the largest and most continuous, it is selected as the pointer mask to eliminate noise from non-pointer pixels.
(2) The pointer skeleton is extracted from the pointer mask image, refining the pointer mask into a row of unit-level pixels. This step facilitates the accurate detection of the pointer’s position and direction.
(3) The Hough transform is applied to detect lines in the processed image. The parameters used are as follows: distance resolution is 1, threshold is 10, minimum line length is 10, and maximum line distance is 400. If a line is detected, each line is traversed to extract the endpoint coordinates (*x*_1_, *y*_1_ and *x*_2_, *y*_2_). These endpoints are then used to draw the corresponding line on the original image, with the endpoint coordinates stored in point1 and point2. If no line is detected, the process returns the message “Cannot detect pointer”.
(4) Calculating the Euclidean distances from point1 and point2 to the image’s center point. The calculation formula is as follows.

(12) $$d=\sqrt{{\left(x_{2}-x_{1}\right)}^{2}+{\left(y_{2}-y_{1}\right)}^{2}},$$

To determine the pointer’s direction, calculate the Euclidean distances from each endpoint (point1 and point2) to the center of the pointer meter image. The point closer to the center is identified as the starting point of the pointer line, while the farther point serves as the endpoint, thus establishing the pointer’s direction.

Traditional methods require the segmentation network to output mask maps for all scales. In contrast, this paper requires only mask images of the first two main scales. A center point extraction algorithm is proposed based on these mask images to accurately determine the specific positions of the scales. The steps for the center point extraction algorithm based on the mask map are as follows:

(1) Determine the scale area: Use the scale mask image from PM-SwinUnet and perform connected component analysis to identify connected areas in the mask image. Traverse each connected area, and if the pixel count is below the threshold n, set these pixels to 0. The remaining connected regions are set to 1, producing the filtered binarized image.
(2) Extract scale contours: Apply OpenCV’s contour detection algorithm to the binary image to extract the scale contours. Save the detected contours to a list for further processing.
(3) Locate scale center points: Iterate through each contour in the list and calculate the minimum enclosing rectangle for each contour. Extract the coordinates of the center point of the smallest enclosing rectangle, convert them to an integer type, and save them to “scale_point”. If the “scale_point” list is empty, return “Cannot detect scale”.

### 3.4. Pointer Meter Reading Recognition

**Figure 7:**
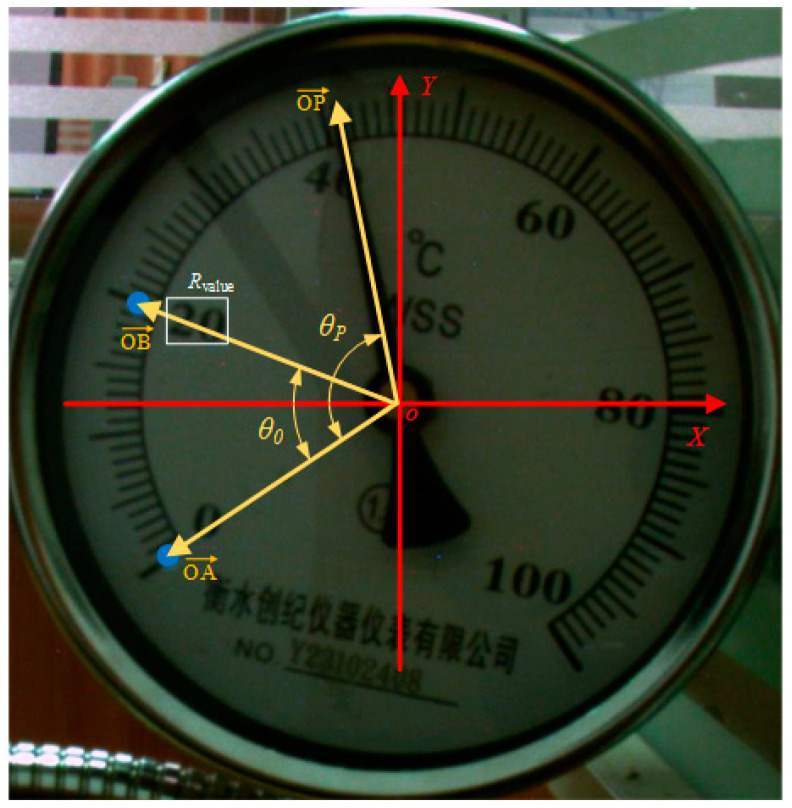
Schematic diagram of pointer meter reading coordinate system.

An improved angle method is proposed to calculate the pointer meter reading based on the pointer and scale information obtained in Section 2.3. To ensure accurate pointer meter readings, a Cartesian coordinate system is established, as illustrated in Figure 7. In this system, the center O of the meter panel is the origin. $\vec{OP}$ represents the pointer, $\vec{OB}$ represents the ray from point O to the second scale, and $\vec{OA}$ represents the ray from point O to the 0 scale. The angles between ray $\vec{OA}$ and rays $\vec{OP}$ and $\vec{OB}$ are denoted as *θ*_0_ and *θ_P_*, respectively. The formula for calculating the included angle is as follows:

(13) $$\theta =\arccos(\frac{{\vec{v}}_{1}\cdot {\vec{v}}_{2}}{\left|{\vec{v}}_{1}\right|\cdot \left|{\vec{v}}_{2}\right|}),$$

where ${\vec{v}}_{1}$ and ${\vec{v}}_{2}$ represent the corresponding vectors, $\left|{\vec{v}}_{1}\right|$ and $\left|{\vec{v}}_{2}\right|$ represent the lengths of the two vectors. Then, convert the included angle from radians to degrees. Then, convert the included angle from radians to degrees. After calculating the specific values of *θ*_0_ and *θ_P_* according to the above formula, the improved angle method can be calculated with the following formula:

(14) $$Reading=\frac{R_{value}}{\theta_{o}}\times \theta_{p},$$

The *R_value_* represents the reading on the second scale, calculated by the Scale Value Recognition Module depicted in Figure 4b. The Scale Value Recognition Module comprises multiple Conv Layers and a connected temporal classification (CTC) [42], enabling scale value recognition via parallel linear prediction. Specifically, a linear classifier with N nodes generates transcript sequences of size W/4. Ideally, identical characters in the component CTC are transcribed as repeated characters, while non-text components are represented as whitespace symbols. The resulting sequence is then automatically compressed into the corresponding numerical value. Here, N is set to 11.

## 4. Results

For pointer meter images captured by industrial cameras, inspection robots, and surveillance cameras, the dials are detected using a target detection algorithm. Subsequently, for a single image of the meter panel, the scale, scale value, and pointer pixel area are extracted through an image segmentation algorithm. Finally, the pointer fitting and meter reading calculation are performed.

### 4.1. Datasets

**Figure 8:**
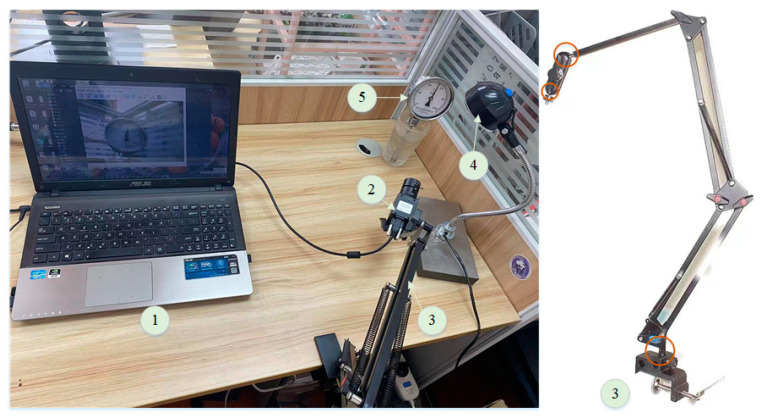
Pointer meter image collection platform (1. laptop computer, 2. industrial cameras, 3. camera brackets, 4. light sources, 5. industrial pointer meters). The left side shows the camera bracket, and the part marked with a circle can rotate 360 degrees.

To accurately detect and identify industrial pointer meters in complex scenes, we developed a pointer meter image collection platform, as shown in Figure 8. The platform consists of a laptop computer (ASUS, Taiwan, China), a camera stand, a light source, an industrial camera (Huatengweishi Technology Co., Ltd, Shenzhen, China), and an industrial pointer meter (Shanghai Saitu Instrument Co., Ltd, Shanghai, China). The industrial camera used is Huatengweishi’s HT-UBS300C-T. This camera is USB-powered and does not require an external power supply. The camera has a maximum resolution of 3 million pixels. The camera stand features an all-metal self-positioning structure, allowing for the camera to be positioned multi-directionally. During image collection, the surrounding environment was adjusted by modifying the position of the industrial thermometer, camera, and light source brightness. HuaTengVision softwareV1.0 was utilized to collect and store the meter images. The collected pointer meter images include conditions such as blur, tilt, overexposure, and darkness. A total of 400 images were collected for each condition, including normal conditions, resulting in a total of 2000 m images. This dataset is named the Real Pointer Meter (RPMeter) Dataset. All pointer meter images were manually annotated using LabelImg and Labelme. To verify the generalization and robustness of the proposed method, experiments were conducted on two public Meter Challenge (MC1296) [19] and Paddle Meter Datasets. The MC1296 dataset contains 1296-m images with complex backgrounds, multiple scales, and various viewing angles captured by automated robots in industrial scenes. The Paddle Meter Dataset contains 1197 pointer meter images, some collected by surveillance cameras in industrial scenes and others by handheld cameras in open environments. These two datasets compensate for the shortcomings of the RPMeter dataset, which does not include meter images from industrial scenes. The effectiveness of the proposed method can be more accurately verified through experiments on the two datasets.

#### Data Enhancement

Deep learning algorithms excel in automatically extracting important features from data, but they typically require large amounts of training data. In cases of insufficient data, data augmentation is often used during the training phase. Common data augmentation methods can be divided into spatial transformations, color distortions, and information loss. For training the detection model in this study, the Mosaic [43] data augmentation method was utilized. This method combines four training images into one image based on a specific ratio. The specific steps are as follows: (1) Randomly select four training images; (2) Flip and scale the four images or change the brightness, saturation, and hue of the original images; (3) Proportionally combine the four images into one. When training the segmentation model, basic data augmentation techniques are applied, such as random cropping, random rotation, and random adjustments to brightness and contrast.

### 4.2. Experiment Details and Evaluation Indicators

#### 4.2.1. Experiment Details

The training environment is based on the PyTorch framework (PyTorch 1.7) in this study. All models were trained in the same environment. The training environment includes Intel Xeon(R) W-2145 @ 3.7 GHz CPU, NVIDIA Quadro RTX 4000 GPU, Ubuntu 20.04, and CUDA 11.2. The input image size for YOLOX-DC is 640 × 640. During training, stochastic gradient descent (SGD) was used to optimize the YOLOX-DC parameters, with a momentum of 0.9 and a weight decay of 0.0005. The initial learning rate was set to 0.001, the batch size was 2, and the training duration was 30 epochs. The scale range for Mosaic data augmentation was (0.1, 2). The input image size and patch size for PM-SwinUnet were set to 224 × 224 and 4, respectively. Weights pre-trained on ImageNet were used to initialize the model parameters. During training, the initial learning rate of the model was 0.001, the batch size was 1, and the training duration was 50 epochs. The SGD optimizer was used for backpropagation, with a momentum of 0.9 and a weight decay of 0.0001.

#### 4.2.2. Evaluation Indicators

The evaluation indicators in this study primarily assess algorithm performance and meter reading accuracy. To evaluate the detection performance of the algorithm on the pointer meter image dataset, precision, recall, inference time (ms), and FPS (number of image frames processed per second) are used as evaluation metrics. The formulas for calculating precision and recall are as follows:

(15) $$Precision=\frac{TP}{TP+FP},$$

(16) $$Recall=\frac{TP}{TP+FN},$$

To evaluate the segmentation performance of the algorithm on dial elements, mean Intersection over Union (mIoU) and mean Pixel Accuracy (mPA) are used as evaluation metrics.

(17) $$\mathrm{mIoU}=\frac{1}{k+1}\sum_{i=0}^{k}\frac{TP}{TP+FP+FN},$$

(18) $$\mathrm{mPA}=\frac{1}{k+1}\sum_{i=0}^{k}\frac{TP}{TP+FP},$$

where *TP* indicates the true positive values, *FP* represents the false positive values and *FN* presents the false negative values.

For the accuracy of meter reading results, this study uses relative error *δ* and reference error *γ* as evaluation metrics. The relative error *δ* measures the error rate between the pointer reading and the true reading. The quoted error *γ* is a commonly used method to express error in meters. It represents the error relative to the full scale of the meter. The relative error *δ* and quoted error *γ* are calculated as follows:

(19) $$\delta =\frac{\left|R_{T}-R_{P}\right|}{R_{T}},$$

(20) $$\gamma =\frac{\left|R_{T}-R_{P}\right|}{R_{M}}$$

Here, *R_T_* is the actual reading marked manually, *R_P_* is the reading predicted by the algorithm, and *R_M_* is the maximum scale value of the meter.

### 4.3. Pointer Dial Detection Comparison Experiment

**Figure 9:**
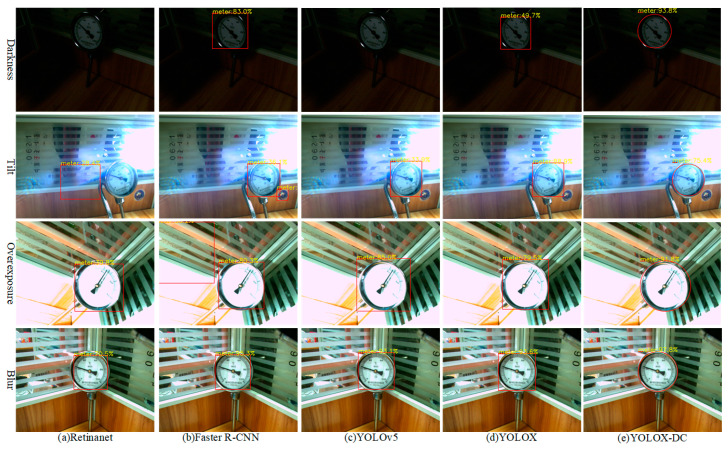
Each column represents the dial detection result of the different algorithms. (**a**) Retinanet. (**b**) Faster R-CNN. (**c**) YOLOv5. (**d**) YOLOX. (**e**) YOLOX-DC.

In this experiment, to verify that the decoupled circle head can detect the pointer meter dial more accurately than the rectangular detection head, YOLOX-DC with a decoupled circle head was compared with four algorithms using rectangular detection heads: Retinanet, Faster R-CNN, YOLOv5, and YOLOX. The detection results of these methods on the RPMeter dataset are shown in Figure 9. Retinanet and YOLOv5 failed to detect the dial under low-light conditions, leading to missed detections. Additionally, Retinanet and Faster R-CNN incorrectly identified other regions in the images as dials, causing false detections. Although YOLOX detects dials in all images, its confidence level was significantly lower compared to YOLOX-DC. The YOLOX-DC algorithm demonstrates several improvements over the above algorithms. The decoupled circle head enables YOLOX-DC to focus on the circular features of the pointer meter dial, improving detection accuracy and reducing background noise in the bounding box. YOLOX-DC consistently shows higher confidence levels in detecting the pointer meter dial across all challenging conditions, indicating its robustness and reliability. Whether under darkness, tilt, overexposure, or blur, YOLOX-DC consistently outperforms the other algorithms, highlighting its ability to generalize across different challenging scenarios. These results demonstrate that the proposed YOLOX-DC algorithm not only accurately detects the dial but also achieves high detection accuracy. Notably, for the circular pointer meter dial, the YOLOX-DC detection results contain less background noise than those of the algorithm with a rectangular detection head.

Secondly, this experiment evaluates and compares YOLOX-DC with four other algorithms using Precision, Recall, and inference time. The detection results on the RPMeter test set are summarized in Table 1. The results indicate that YOLOX-DC achieves a precision rate of 0.996 and a recall rate of 0.997 under IoU_50:95_, higher than the other four algorithms. Although YOLOX-DC’s inference time is 0.04 ms slower and its FPS is 0.36 lower than YOLOX, its precision and recall rates are 3.2% and 2.4% higher, respectively. This positions YOLOX-DC as the most balanced and effective method, combining state-of-the-art accuracy with near-real-time processing capabilities. These advantages make it particularly suitable for industrial applications requiring precise and reliable detection under challenging conditions. Additionally, the experimental results further verify that the decoupled circular head has higher accuracy in detecting pointer meter dials than the rectangular detection head.

**Figure 10:**
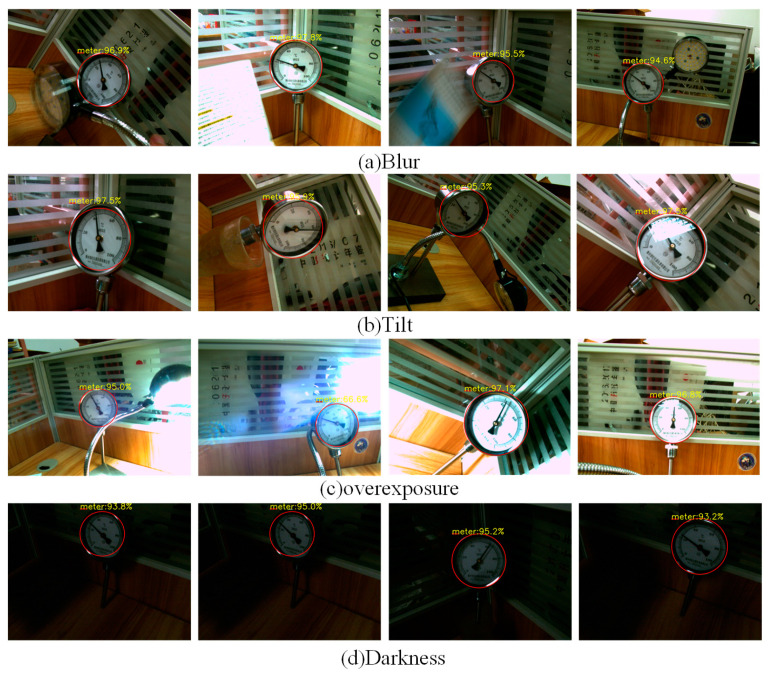
YOLOX-DC dial detection results on low-quality meter images.

**Figure 11:**
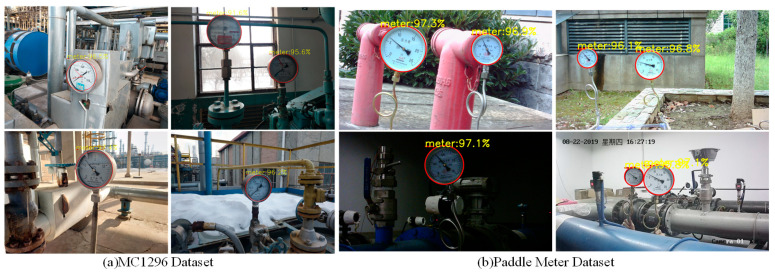
YOLOX-DC dial detection results on MC1296 and Paddle Meter dataset.

To verify the robustness and generalization of the proposed method. First, detection accuracy is assessed using meter images with various challenging conditions from the RPMeter dataset, including blurred, tilted, overexposed, and dark images. The detection results of the dials under these conditions are shown in Figure 10. As illustrated, the proposed YOLOX-DC algorithm not only achieves high detection accuracy but also effectively avoids detecting dial-like objects in the meter image, demonstrating its robustness against diverse environmental challenges. Second, experiments were conducted on the MC1296 Dataset and Paddle Meter Dataset, with the detection results shown in Figure 11. These results indicate that YOLOX-DC performs well in handling meter images captured in various complex situations, such as images with multiple scales, diverse viewing angles, and intricate backgrounds. Therefore, these experiments demonstrate the effectiveness, robustness, and generalization capabilities of the YOLOX-DC algorithm in accurately detecting pointer meter dials across diverse scenarios.

### 4.4. Dial Element Segmentation Experiment

**Figure 12:**
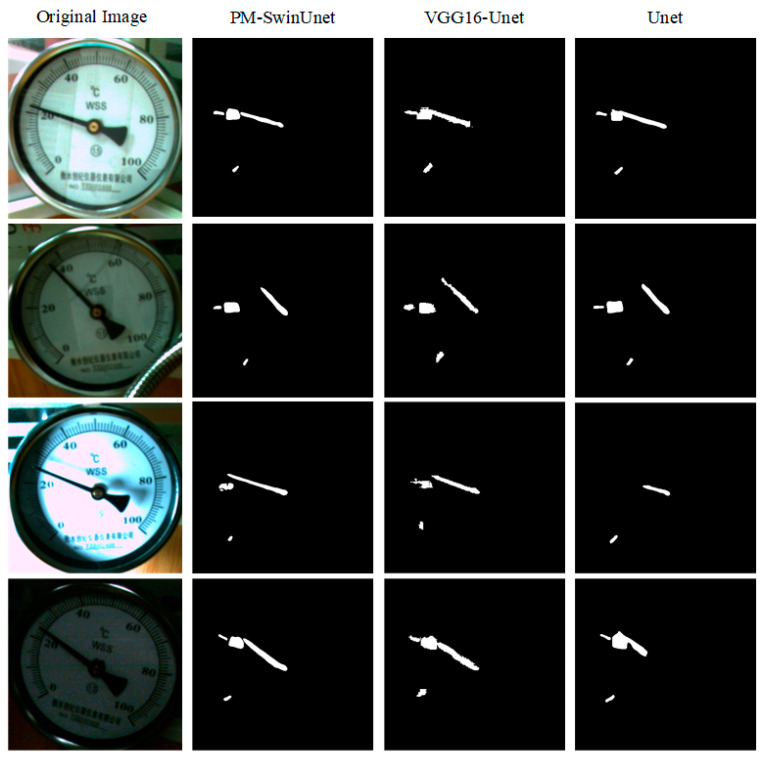
Segmentation results of pointers, scales and scale values by different image segmentation networks.

In this experiment, the input of the PM-SwinUnet segmentation network is 224 × 224 × 3. After using the YOLOX-DC dial detection method, 1966 dial images were obtained through the minimum circumscribed rectangle. Of these, 1600 images were used for training and 366 images for evaluation. In order to verify the effectiveness of the proposed PM-SwinUnet image segmentation network. We built the same experimental environment to compare the PM-SwinUnet with the VGG16-Unet and U-Net models. The comparison results are shown in Figure 12. For dials under different lighting, overexposure, and image blur, the PM-SwinUnet network achieved better segmentation results. Although the VGG16-Unet segmentation network can correctly fit the scale and pointer areas, the segmented results have small jagged edges, and some segmented areas are not separated. Similarly, the U-Net segmentation network suffers from regional connectivity issues and fails to detect certain scales or completely segment pointers under overexposure conditions. In contrast, the PM-SwinUnet network produces segmentation results with smooth edges, no regional connectivity issues, and no missed scale detections or incomplete pointer segmentations, as shown in the Figure 12. These experiments’ results demonstrate that the PM-SwinUnet meter image segmentation network, based on the UNnet-like Swin Transformer, effectively addresses the challenges of detecting small-scale objects and incompletely segmenting long objects. This improvement can be attributed to the Swin Transformer Block, which integrates the window-based multi-head self-attention (W-MSA) features of the previous layer with the shifted window multi-head self-attention (SW-MSA) features of the next layer. This mechanism enables the calculation of global features and long-dimensional information, significantly enhancing segmentation accuracy and robustness.

In addition to visualizing the segmentation results, this experiment evaluates and compares three segmentation networks using mPA and mIoU. The comparison results are presented in Table 2. The table shows that the mPA and mIoU of PM-SwinUnet reached 0.976 and 0.941, respectively. Compared with Unet’s mPA and mIoU, PM-SwinUnet improved by 2.9% and 3.3%, respectively. Compared to other methods, PM-SwinUnet integrates the features extracted by Swin Transformer into the decoder stage of U-Net, further enhancing its ability to fuse multi-scale features. Small-scale objects benefit from the encoder capturing their overall contours, while the decoder refines their edge features using local attention. Moreover, extracting features for elongated objects requires capturing their global structure, which is facilitated by Swin Transformer’s global attention mechanism. In contrast, traditional convolutional kernels operate locally and struggle to capture the complete structure of elongated objects. By integrating U-Net, PM-SwinUnet leverages multi-scale context to enhance the representation of elongated objects. Therefore, the above experimental results verify that the combination of Swin Transformer and U-Net network architecture significantly enhances the feature extraction capabilities of the PM-SwinUnet segmentation network for small-scale and long-term objectives.

### 4.5. Scale and Pointer Fitting Experiment Results

In the previous segmentation experiment, although the mask images of scales and pointers were accurately segmented, further fitting of the scale and pointer pixel areas is required. To address this, this article proposes the Scale and Pointer Fitting Module. Given the large number of pointer pixels, fitting all pixels would be computationally expensive and time-consuming. To optimize this process, the pointer skeleton is extracted from the pointer mask image using a skeleton thinning algorithm, significantly reducing the number of pixels requiring calculation. Additionally, a center point extraction algorithm is introduced to determine the precise location of the scale. This algorithm operates on the scale mask map, ensuring accurate localization of the scale’s center points.

**Figure 13:**
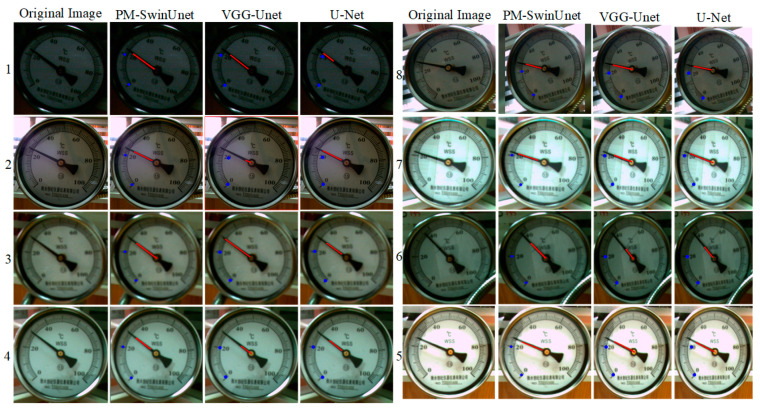
Comparison of scale and pointer fitting results of different image segmentation networks.

In order to verify the effectiveness of the Scale and pointer fitting module, it was applied to different image segmentation networks, with comparison results shown in Figure 13. Experimental results demonstrate that for VGG-Unet and U-Net segmentation networks with poor performance, the proposed Scale and pointer fitting module remains highly robust, fitting scales and pointers well. Additionally, for defective pointer pixel areas, the pointer can still be fitted well. For instance, in images number 1 and 6, segmented by the U-Net network, the pointer can still be accurately fitted despite incomplete segmentation in the pointer region. Furthermore, Figure 13 shows that the meter image segmented by PM-SwinUnet, when combined with the Scale and Pointer Fitting Module, achieves more precise scale and pointer fitting. These experimental results further validate that the combination of Swin Transformer and U-Net network architecture significantly enhances the feature extraction capabilities of the PM-SwinUnet segmentation network, particularly for small-scale and elongated objects.

### 4.6. Pointer Reading Recognition Comparison Experiment

**Figure 14:**
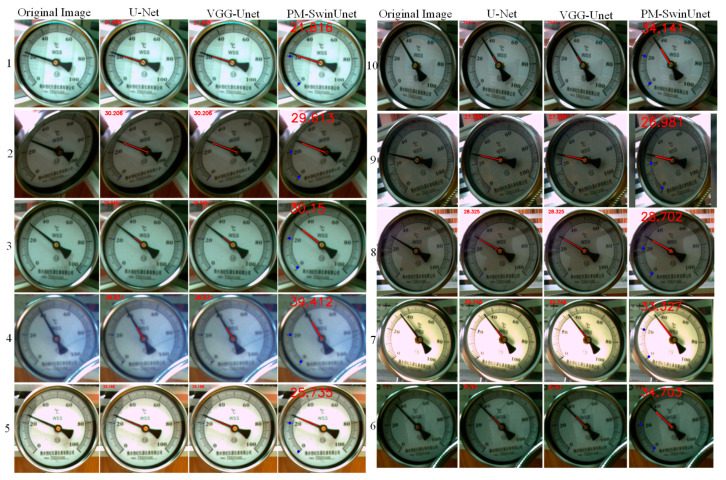
Comparison of reading results of improved angle method in different image segmentation networks.

The robustness of the proposed reading identification method was evaluated using different image segmentation networks, and the results were compared with manual readings. For manual readings, according to pointer meter specifications, the reading should be controlled at 1/5 of the minimum scale. As shown in Figure 14, the original meter image and the results from the three segmentation networks are presented in sequence from columns 1 to 8, showing the results of pointer fitting, scale fitting, and reading recognition. The red numbers in Figure 14 indicate the pointer meter readings recognized by the improved angle method. Detailed results are shown in Table 3. The table shows that the proposed recognition method adapts well to the segmentation results of different image segmentation networks, achieving an average relative error of 0.0092 and an average citation error of 0.0026. Compared to VGG-Unet and U-Net networks, the proposed method demonstrates significant improvements in accuracy. The maximum error between the proposed method’s identified reading and manual reading is 0.793 °C. Given the thermometer’s error accuracy of ±1 °C used in this experiment, this error level is within a reasonable range.

**Figure 15:**
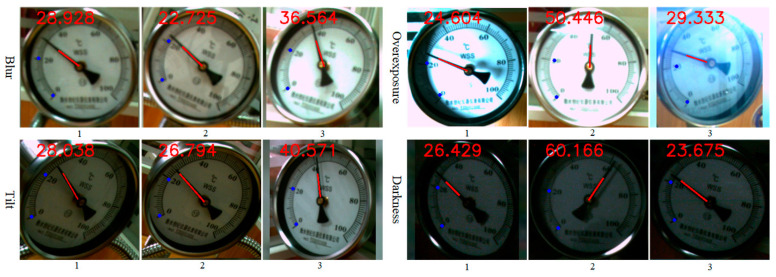
Recognition results of low-quality meter images.

In order to verify the performance of the proposed method in processing low-quality meter images, recognition results of four types of low-quality meter images, including blur, tilt, overexposure and dark light, were compared. Figure 15 shows the recognition results of low-quality meter images. As shown, the scale and pointer fit well, and the recognized readings are within the error range. Notably, the proposed method can accurately identify readings even in overexposed images. Detailed recognition results for low-quality meter images are summarized in Table 4. The maximum relative errors for blurred, tilted, overexposed, and low-light images were 0.0093, 0.0523, 0.0039, and 0.0094, respectively. The maximum reference errors were 0.0027, 0.0133, 0.0009, and 0.0057, respectively. Compared to the allowable error of ±0.01, recognition errors for all low-quality images except tilted ones are within the acceptable range. Although the proposed method’s performance decreases for tilted images, it remains highly accurate for most normal meter images and demonstrates excellent robustness in identifying readings under challenging conditions.

**Figure 16:**
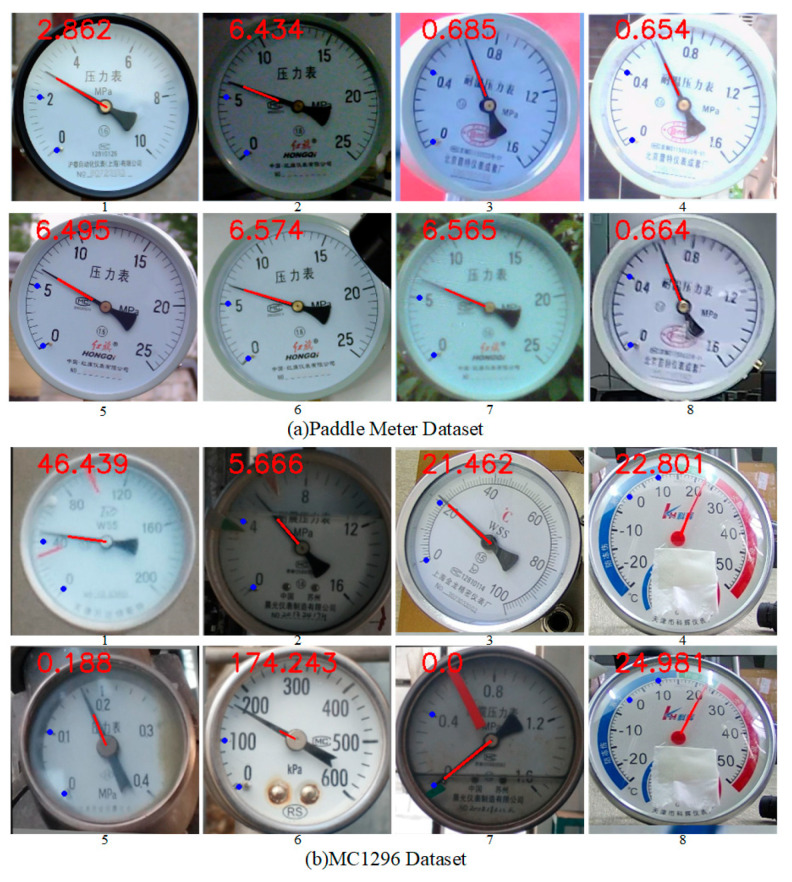
Pointer, scale fitting and reading recognition results of Paddle Meter Dataset and MC1296 Dataset.

To verify the generalization of the proposed method, experiments were conducted on the public Paddle Meter Dataset and MC1296 Dataset. The test results are presented in Figure 16. The proposed method accurately fits the pointers and scales of various meters and correctly identifies meter readings. Detailed meter reading results are presented in Table 5, where the maximum reference errors for the three meters in the Paddle Meter Dataset are 0.0022, 0.0030, and 0.0087, respectively. The allowable errors for the three meters in the Paddle Meter Dataset are 0.02, 0.02, and 0.03, respectively. The quoted error of the proposed method is significantly lower than the allowable error. The MC1296 Dataset contains 7-m types, with the reference error of image No 5 reaching 0.025. Compared to its allowable error of 0.05, this reference error is within reasonable range. These experimental results demonstrate that the proposed method exhibits strong generalization performance, accurately identifying meter readings across diverse datasets and meter types.

Time efficiency is a fundamental requirement for any automatic detection and recognition system. This paper gives the time consumed by each process when calculating the pointer meter reading, as detailed in Table 6. The table shows that the process of segmenting the dial elements takes more time. However, this is justified because the performance of scale and pointer segmentation is crucial to the accuracy and robustness of the subsequent pointer meter reading calculations. Furthermore, a comparison of time consumption between the proposed method and other methods is provided in Table 7. The results indicate that the proposed method consumes significantly less time in both the detection and recognition phases compared to other methods. Notably, the overall time consumption of the proposed method is twice as fast as that reported in the literature [32]. The above comparison experiments show that the proposed method has the advantage of quickly identifying meter readings, which can provide strong support for the digital transformation of the manufacturing industry.

## 5. Discussion

This study demonstrates the superior performance and robustness of the proposed YOLOX-DC and PM-SwinUnet frameworks in pointer meter detection, segmentation, and reading recognition. YOLOX-DC, with its decoupled circular head, effectively focuses on circular features, achieving higher precision and recall than traditional methods, even under challenging conditions like low light, tilt, and overexposure. Similarly, PM-SwinUnet leverages Swin Transformer blocks to capture local and global features, significantly improving segmentation accuracy for small-scale and elongated objects. The Scale and Pointer Fitting Module further enhances segmentation results by optimizing pointer fitting with skeleton thinning and center point extraction. Integrated with PM-SwinUnet, it achieves precise scale and pointer localization, even for noisy or incomplete data. The improved angle method achieves an average relative error of 0.0092, demonstrating robust reading recognition across various imaging conditions and datasets. Finally, the proposed approach outperforms existing methods in time efficiency while maintaining accuracy, underscoring its potential for industrial applications.

## 6. Conclusions

Pointer meter reading recognition is a key component in the digital transformation of the manufacturing industry. However, variations in illumination intensity and shooting angles often lead to low accuracy and poor robustness in existing methods for reading pointer meters. This paper proposes a YOLOX-DC dial detection algorithm and a PM-SwinUnet image segmentation network to address these challenges. First, the pointer meter dial is detected using the YOLOX-DC network. The decoupled circle head is more in line with the contour characteristics of the pointer meter and can detect the meter dial more accurately than the rectangular detection head. Second, the PM-SwinUnet image segmentation network is used to obtain the mask map of the scale lines, scale value, and pointer. These mask maps are combined with the skeleton thinning algorithm and center point extraction algorithms in the scale and pointer fitting module to accurately fit the meter pointer and the first two main scales of the dial. Finally, the improved angle method uses the relationship between the pointer’s rotation angle and the first two main scales to achieve accurate readings. The robustness and generalization of the proposed method are verified through a series of experiments. Experimental results show that the YOLOX-DC algorithm improves dial detection accuracy. On the RPMeter dataset, its accuracy and recall rates reached 99.6% and 99.7%, which are 3.2% and 2.4% higher than the YOLOX algorithm, respectively. The average inference time for a single-meter image was 7.11 ms. The mIoU and mPA of the PM-SwinUnet segmentation network reached 94.1% and 97.6%, respectively. The average relative error and average reference error of the reading calculation method are 0.0092 and 0.0026, respectively, significantly lower than the allowable error. The time consumption to identify a meter image is only 0.5982 s. Therefore, the experimental results illustrate that the proposed method is capable of realizing high accuracy and fast recognition of pointer meter readings in real industrial environments. It can also show that the proposed method provides a referable solution for the digital transformation of the manufacturing industry.

However, the proposed method also has limitations. For example, the proposed method is not applicable to rectangular or irregularly shaped pointer meters in the detection phase. Moreover, the proposed method has multiple computational steps in the recognition phase, such as semantic segmentation, pointer fitting, and reading calculation. In order to meet the requirements of real-time and low computational resources in practical applications, optimizing the complex reading calculation process and reducing the computational complexity is the focus of future research. Meanwhile, the generalization performance of the proposed method will be improved so that it can detect other shapes of pointer meters.

## Figures and Tables

**Table 1:** Comparative results of five detection algorithms under IoU_50:95_.

Methods	Precision	Recall	Time (ms)	FPS
Retinanet	0.923	0.947	17.16	18.19
Faster R-CNN	0.945	0.959	53.11	16.38
YOLOv5	0.959	0.968	30.49	19.64
YOLOX	0.964	0.973	6.97	24.42
YOLOX-DC(ours)	0.996	0.997	7.11	24.06

**Table 2:** Comparison results of mIoU and mPA of different segmentation.

Method	mIoU	mPA
VGG16-Unet	0.925	0.963
U-Net	0.908	0.947
PM-SwinUnet	0.941	0.976

**Table 3:** Error comparison results when the improved angle method is applied to different image segmentation methods.

No	Meter Reading				*δ*			*γ*		
	Real	Proposed	VGG-Unet	U-Net	Proposed	VGG-Unet	U-Net	Proposed	VGG-Unet	U-Net
1	22.40	21.816	21.855	21.918	0.0260	0.0243	0.0215	0.0058	0.0054	0.0048
2	28.82	29.613	30.206	30.623	0.0275	0.0481	0.0628	0.0079	0.0138	0.0181
3	30.10	30.150	29.945	30.756	0.0017	0.0051	0.0218	0.0001	0.0015	0.0066
4	40.00	39.412	38.831	43.13	0.0147	0.0292	0.0828	0.0058	0.0117	0.0331
5	25.84	25.735	25.166	24.884	0.0041	0.0261	0.0369	0.0010	0.0067	0.0095
6	35.10	34.703	35.792	34.623	0.0113	0.0197	0.0136	0.0039	0.0069	0.0048
7	33.52	33.327	33.746	34.449	0.0057	0.0067	0.0277	0.0019	0.0023	0.0093
8	28.70	28.702	28.325	28.467	0.0000	0.0131	0.0081	0.0000	0.0037	0.0023
9	26.98	26.981	27.296	27.359	0.0000	0.0117	0.0140	0.0000	0.0032	0.0038
10	34.16	34.141	33.940	33.249	0.0005	0.0064	0.0266	0.0002	0.0022	0.0091
Avg	—	—	—	—	0.0092	0.0190	0.0316	0.0026	0.0057	0.0101

**Table 4:** Meter recognition errors for blur, tilt, overexposure, and darkness.

Group	No	Real	Proposed	*δ*	*γ*
Blur	1	29.20	28.928	0.0093	0.0027
	2	22.58	22.725	0.0064	0.0015
	3	36.36	36.564	0.0056	0.0020
Tilt	1	26.98	28.038	0.0059	0.0016
	2	25.46	26.794	0.0523	0.0133
	3	41.10	40.571	0.0129	0.0053
Overexposure	1	24.70	24.604	0.0039	0.0009
	2	50.38	50.446	0.0013	0.0007
	3	29.36	29.333	0.0009	0.0003
Darkness	1	26.42	26.429	0.0003	0.0000
	2	60.64	60.166	0.0094	0.0057
	3	23.66	23.675	0.0006	0.0002

**Table 5:** Meter reading recognition results from public datasets.

Dataset	No	Real	Proposed	δ	γ
Paddle Meter Dataset	1	2.84	2.862	0.0077	0.0022
	2	6.42	6.434	0.0022	0.0006
	3	0.68	0.685	0.0073	0.0031
	4	0.64	0.654	0.0218	0.0087
	5	6.48	6.495	0.0023	0.0006
	6	6.50	6.574	0.0113	0.0030
	7	6.56	6.565	0.0008	0.0002
	8	0.66	0.664	0.0061	0.0025
MC1296 Dataset	1	46.34	46.439	0.0032	0.0008
	2	5.66	5.666	0.0011	0.0004
	3	21.48	21.462	0.0008	0.0002
	4	22.86	22.801	0.0026	0.0011
	5	0.178	0.188	0.0561	0.0250
	6	169.86	174.243	0.0258	0.0073
	7	0.00	0.0	0.0000	0.0000
	8	22.98	24.981	0.0870	0.0191

**Table 6:** Time consumption for each processing step of the proposed method.

Process	Time (ms)
Detecting dial	7.10
Segmenting dial element	305.9
Fitting scale and pointer	183.0
Calculating meter reading	2.2

**Table 7:** Comparison of time consumption of the proposed method with other methods.

Method	Detection Time (ms)	Recognition Time (ms)	Total Time (ms)
Zhai et al. [5]	530	-	-
Gao et al. [44]	-	3512	-
Ji et al. [33]	534	1037	1571
Ours	7.1	591.1	598.2

## Data Availability

The data presented in this study are available on request from the corresponding author.
